# The Influence of Shape on the Output Potential of ZnO Nanostructures: Sensitivity to Parallel versus Perpendicular Forces

**DOI:** 10.3390/nano8050354

**Published:** 2018-05-22

**Authors:** José Cardoso, Filipe F. Oliveira, Mariana P. Proenca, João Ventura

**Affiliations:** IFIMUP-IN, and Department of Physics and Astronomy, Faculty of Sciences, University of Porto, Rua do Campo Alegre, 687, 4169-007 Porto, Portugal; filipe.f.oliveira91@gmail.com (F.F.O.); marianapproenca@gmail.com (M.P.P.)

**Keywords:** ZnO, nanowires, piezoelectric nanogenerators, energy harvesting, numerical simulations, nanomushrooms, nanotrees

## Abstract

With the consistent shrinking of devices, micro-systems are, nowadays, widely used in areas such as biomedics, electronics, automobiles, and measurement devices. As devices shrunk, so too did their energy consumptions, opening the way for the use of nanogenerators (NGs) as power sources. In particular, to harvest energy from an object’s motion (mechanical vibrations, torsional forces, or pressure), present NGs are mainly composed of piezoelectric materials in which, upon an applied compressive or strain force, an electrical field is produced that can be used to power a device. The focus of this work is to simulate the piezoelectric effect in different ZnO nanostructures to optimize the output potential generated by a nanodevice. In these simulations, cylindrical nanowires, nanomushrooms, and nanotrees were created, and the influence of the nanostructures’ shape on the output potential was studied as a function of applied parallel and perpendicular forces. The obtained results demonstrated that the output potential is linearly proportional to the applied force and that perpendicular forces are more efficient in all structures. However, nanotrees were found to have an increased sensitivity to parallel applied forces, which resulted in a large enhancement of the output efficiency. These results could then open a new path to increase the efficiency of piezoelectric nanogenerators.

## 1. Introduction

From the latest reports on nanogenerators’ development [1,2], one can finally fabricate permanent power sources that constitute a new type of renewable and clean energy. These nanogenerators can now be implemented in many technological devices because, as the components’ size decreased, so too did their power consumptions. Nanogenerators (NGs) that provide power in the micro- to milli-Watt range are, thus, suitable as self-powered systems. NGs will change our lives and the way we use our everyday electronics, increasing the number of independent devices, which opens a new industrial path that can be very fruitful and important in the future. Their small size and flexibility are huge advantages when it comes to creating remote sensors, such as environmental monitoring equipment and weather control. Its self-sufficient functioning allows for new devices that can be put in otherwise impossible places, with no need to replace, recharge, or maintain them. NGs can also be applied to living beings. With self-powered biosensors, we can perform real time monitoring and faster diagnoses, which will constitute a breakthrough in medical analyses by gathering wireless biometric data emitted by a NG inside us [3].

The natural environment provides us with several different sources of unexplored energy, such as wind oscillation, water streams, sun heating, and living beings’ movement. It also presents us with different materials capable of converting such natural energy into useful electricity, such as thermoelectric [4], triboelectric [5], and piezoelectric [6] materials. These materials will be discussed in turn. Thermoelectric materials allow the production of electricity from a thermal gradient [4,7,8]. However, the thermoelectric efficiency related with its figure of merit ZT is typically limited to unity due to the material’s properties. Furthermore, thermoelectric NGs present important downsides when miniaturized, including a difficulty in maintaining a high temperature difference between the two ends. Triboelectric materials possess the property of becoming electrically charged upon friction, contact, or adhesion with another triboelectric material, creating positive or negative charges depending on the material’s tendency to gain or lose electrons. It was recently demonstrated that when two triboelectric materials with different polarities come into contact, useful electrical power can be generated [5,9,10,11]. Although highly efficient, their working configurations are limited by the necessity of contact or friction between two different materials.

On the other hand, piezoelectric NGs rely on the piezoelectric effect, which is the creation of an electric potential by means of deformation of a material, or vice-versa. Applying a tensile or compressive force to a non-centrosymmetric crystalline material leads to the displacement of the positive and negative centers of charge, inducing a piezoelectric polarization and oppositely charged surfaces in the material. Electrical energy is then obtained from the piezoelectric material and injected in an external circuit. The first reported piezoelectric NG [12] was composed of an array of ZnO nanowires (NWs) and an atomic force microscope with a platinum tip was used to deform the NWs, creating a piezoelectric potential. In this device, a Schottky barrier, i.e., a metal–semiconductor interface that acts as a diode to the electric current, was created at the point of contact between the Pt tip and the ZnO NWs. A maximum output voltage of 6.5 mV was achieved. This configuration presented crucial limitations, such as low output power density (around 1 mW/cm^2^), a single deformation possibility, and reduced applicability, leading to a search for novel and more efficient configurations [13,14,15,16,17]. These included a piezoelectric NG in which the deformation of the NWs was induced by an array of metallic tips grown on one side of a substrate, while the other side was filled with ZnO NWs, allowing the stacking of several substrates (leading to power densities of up to 0.11 μW/cm^2^) [13]. Vertical (VING) and lateral (LING) nanowire array NGs were also developed [18]. The applications for piezoelectric NGs are extensive because of the possibility to use all flexible and dynamic surfaces, like cloth or shoes, touchable electronics, or even the human body, to produce electricity. As is clear above, the most common material used for the development of piezoelectric NGs is zinc oxide [12,17]. Typically, it can be found in the form of a solid crystal, where it is transparent, or in powder form, where it is white. ZnO is a semiconductor with a wide and direct band-gap in the range of 3.3 to 3.7 eV [19], which helps to create an efficient semiconductor for high power and temperature applications. The highest piezoelectric coefficient (*d*_33_ = 12.3 pm∙V^−1^) of the strain piezoelectric tensor of ZnO is associated with the (002) crystallographic plane [20]. In addition, in optical processes, it provides an ultra-violet photo-luminescence when excited. ZnO has a good mechanical-to-electrical conversion, as it presents a non-centrosymmetric hexagonal wurtzite crystal structure. ZnO can be deposited as thin films (2D), or grown in various nanostructures, such as nanowires, nanotubes or nanorings [21,22], using several techniques, such as electrochemical deposition [23], hydrothermal processes [24], ion beam deposition [25], electrodeposition [26], or sol-gel [27]. Additionally, contrary to lead zirconate titanate (PZT) and lead-based materials, ZnO is a bio-compatible material.

In this work, the finite elements method (FEM) was used to simulate the output potential of various piezoelectric ZnO nanostructures upon deformation, applying lateral and vertical loads on its surfaces. The objective of this study was to understand how one could enhance the output potential of NGs by modifying the nanowire’s morphology and achieve better efficiency for lateral applied forces, which present a considerably lower performance than perpendicular forces. Thus, we decided to create piezoelectric nanostructures in the form of mushrooms [28] and trees [29], aiming to improve the overall efficiency of piezoelectric NGs.

## 2. Numerical Methods

We have implemented a numerical model in COMSOL to study the output potential of nanowire piezoelectric materials [30]. This model, which was validated against the available literature (both numerical and experimental) [15,31,32,33,34,35], was extended here to more complex 3D models and the study of the effects of different properties and behaviors on piezoelectric NGs. To create a single nanowire, we first define its radius (*R*) and height (*H*), and the ratio between *H* and 2*R* gives us the aspect ratio.

To simulate the effects of mechanical loads on surfaces, we start from an equilibrium condition, where there are no body forces ($f_{e}^{\left(b\right)}=0$) [36] acting on our structures, such that:

(1) $$\nabla .\sigma =f_{e}^{\left(b\right)}=0,$$

where σ is the stress tensor, which is related to the strain (ε), electric field (*E*), and electric displacement (*D*) [37], as follows:

(2) $$\{\begin{array}{c} \sigma_{\mu }=c_{\lambda \mu }\varepsilon_{\mu }-e_{i\mu }E_{i}\\ D_{i}=e_{i\mu }\varepsilon_{\mu }+\epsilon_{ij}E_{j} \end{array},$$

In Equation (2), $c_{\lambda \mu }$ is the Young’s modulus, $e_{i\mu }$ the stress piezoelectric coefficient, and $\epsilon_{ij}$ is the permittivity constant, where *i*, *j* = 1, 2, 3 and *λ*, *μ* = 1, 2, 3, 4, 5, 6. To describe an applied force in a random direction, we use $e_{i\mu }$, which for ZnO [38] is given by:

(3) $$e_{i\mu }=\left[\begin{array}{ccc} 0 & 0 & 0\\ 0 & 0 & 0\\ e_{31} & e_{31} & e_{33} \end{array}\;\begin{array}{ccc} 0 & e_{15} & 0\\ e_{15} & 0 & 0\\ 0 & 0 & 0 \end{array}\right],$$

using the Voight’s notation. Additionally, assuming that there are no free charges, we obtain:

(4) $$\nabla .D=\rho_{e}^{\left(b\right)}=0,$$

For the simulations performed in this work, we assumed several conditions. First, the boundary load, i.e., the part of the surface where the force is applied, is always at the top of the wires. The boundary load consists of a force that can be defined as $\sigma .\vec{n}=\frac{{\vec{F}}_{total}}{A}$, where $\vec{n}$ is the vector of the applied force, ${\vec{F}}_{tot}$ is the total applied force and A the area [39]. In our study, we varied the total applied force and studied its influence on the open circuit output voltage. Additionally, note that, as usual in this type of model, the semiconductor physics effects of ZnO are not directly taken into account. The parameters used in the simulations are listed in Table 1 [40].

To properly study our wires, we have to define one boundary as a base, where it has a fixed constraint u=0 that models the structurally blocked face of the system, ground connection, and zero potential (V=0). In this work, this was always located at the bottom of the structure. As in Reference [41], we do not observe an inversion of the generated voltage at the base of the nanowire. The study was then divided into three different steps, with increasing structural complexity. First, a single cylindrical ZnO nanowire (*H* = 500 nm and *R* = 50 nm) was created. Then, we studied the same nanowire in a mushroom form, with a semi-spherical tip with *R* = 100 nm on top. Finally, a nanowire tree was simulated, where from a single cylindrical nanowire (*H* = 500 nm and *R* = 50 nm), several branches were attached (with *R* = 25 nm). These simulations used a tetrahedral mesh, with element sizes between 5.1 and 40.7 nm. All simulations were performed in a stationary mode and the output potential was calculated as a function of the external force (*F*_tot_), varying from 0.01 to 0.1 μN, in 0.005 μN steps, applied along the *x-*, *y-*, and *z-*directions. Here, the same total force was applied in all the top surface boundaries of the nanostructures.

## 3. Results and Discussion

### 3.1. Cylindrical Nanowire

**Figure 1:**
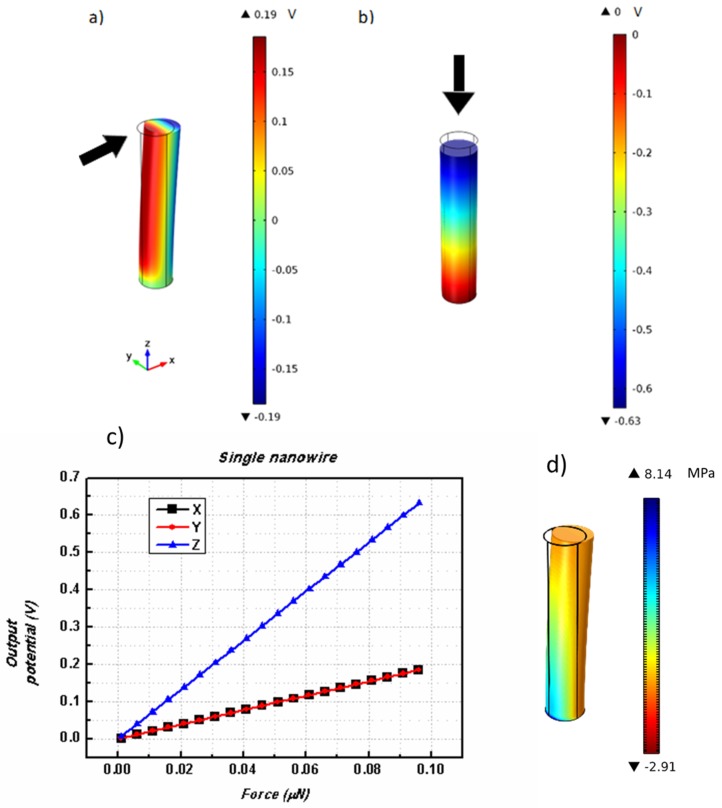
Nanowire structural deflection and corresponding piezoelectric potential when applying (**a**) *F*_x_, and (**b**) *F*_z_, on the top surface. The *c*-axis is directed along the long nanowire direction. (**c**) Resulting piezoelectric output potentials as a function of the total applied force along the *x*-, *y*-, and *z*-directions. A color scale in Volts shows the potential distribution on the nanowire. (**d**) Calculated stress for a force applied along the *x*-direction.

The piezoelectric voltage generated by ZnO nanowires has been a subject of extensive experimental and numerical studies. Therefore, we have benchmarked our results on complex ZnO structures against those obtained in single nanowires. The first study was then performed in a single cylindrical nanowire with *H* = 500 nm and *R* = 50 nm. Deformation forces, *F*_x_, *F*_y_, and *F*_z_, were applied on the top wire surface along the *x-*, *y-,* and *z-*axes, respectively, and the electric potential generated in the nanowire was analyzed. Figure 1a,b illustrate the displacement and piezoelectric potential of the nanowire when applying *F*_x_ and *F*_z_, respectively. On the other hand, Figure 1d shows the calculated stress along the nanowire for a force applied along the *x*-direction. In accordance with the obtained piezoelectric potential, the stress is seen to decrease gradually as one moves from the structurally fixed face of the nanowire, where the largest deformation takes place.

As displayed in Figure 1, the electric potential for the same amount of force is larger for a perpendicular compressive force (*F*_z_) than for a parallel bending force (*F*_x_ and *F*_y_). The plots in Figure 1c show a comparative analysis of the output values obtained for each applied force. To better compare the effectiveness of each force, all measurements of potential are in absolute values. As expected, the output potential increases linearly with the applied force. By analyzing Figure 1b, one can observe that, for a maximum force of 0.1 μN, a maximum potential of 0.63 V is obtained for an *F*_z_ compressive force. However, for the same applied force value along the *x*- and *y*-directions (*F*_x_ and *F*_y_ bending forces), one obtains an output voltage of only 0.19 V. This can be explained by the fact that, for a single ZnO crystal, the piezoelectric coefficient along the vertical axis of the nanowires ($e_{33}$) has the highest value. Therefore, for a force applied along the *c*-axis (the nanowire long axis), we have a more efficient energy conversion and thus a higher electrical potential.

### 3.2. Nanomushroom

**Figure 2:**
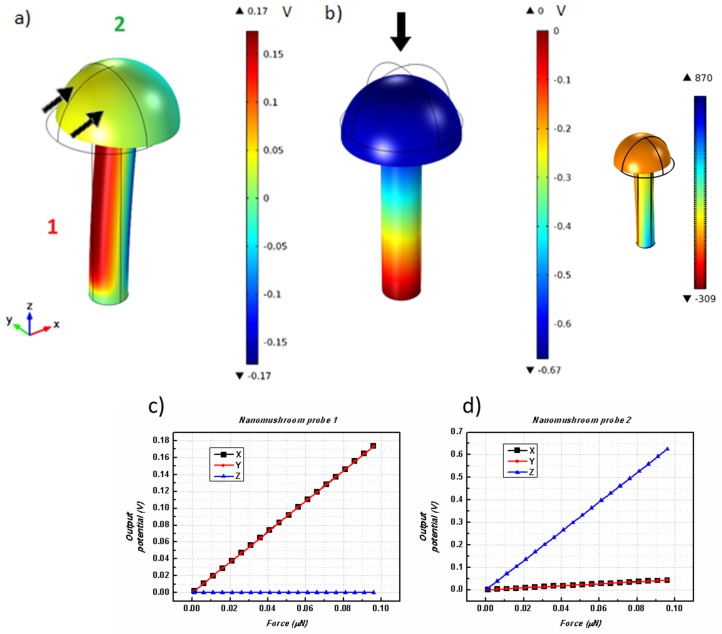
Nanomushroom structural deflection and corresponding piezoelectric potential when applying (**a**) *F*_x_, and (**b**) *F*_z_, on the top surface. The *c*-axis is directed along the long nanowire direction. Inset of (**b**) shows the calculated stress for a force applied along the *x*-direction. (**c**,**d**) Resulting piezoelectric output potentials as a function of the total applied force along the *x*-, *y*-, and *z*-directions for (**c**) probe 1 and (**d**) probe 2. The location of the measurement probes 1 and 2 are represented in (**a**). A color scale in Volts shows the piezoelectric potential distribution on the structure.

As a way to improve the output potential of a piezoelectric ZnO nanostructure, a nanomushroom morphology (Figure 2a) was created. A cylindrical nanowire (*H* = 500 nm, *R* = 50 nm) with a hemisphere on top (with *R* = 100 nm) was created. Theoretically, one expects the hemispherical tip to enhance the lateral bending of the nanostructure because of its higher surface area. With the increased complexity of this structure, we use two probes to observe output potential values in the nanowire base and in the hemispherical tip, as depicted in Figure 2a.

The results obtained for forces applied along the *x*-, *y*-, and *z*-axes are illustrated in Figure 2. Measurements of the output potential were then performed in the wire (probe 1) and on top of the mushroom tip (probe 2). The absolute values of the output potentials for probes 1 and 2 as a function of the applied force in the different axes are illustrated in Figure 2b,c.

Analyzing the results, one can observe that, even though the *F*_x_ and *F*_y_ applied forces were expected to be more efficient in this structure, the obtained output potentials were similar to those achieved with a simple cylindrical nanowire. This effect is due to the higher inertia caused by an increase of mass on the structure. Nevertheless, due to its larger surface area on the top, in a practical situation one can deform this structure easier than a simple nanowire. Confirming these results, the stress distribution obtained along the nanomushroom for a force applied along the *x*-direction is similar to that obtained for a single nanowire (inset of Figure 2b). On the other hand, for a perpendicular *F*_z_ force, an improvement on the output value is verified and the highest outputs are again obtained for the vertical axis along the structure. An output of 0.67 V was determined for a 0.1 μN *z*-axis compressive force, a value that is slightly larger than for a cylindrical nanowire. This can be explained again by the presence of the extra mass formed by the mushroom tip that increases the amount of compression on the nanowire base. As mechanical-to-electrical conversion is a linear effect, larger deformations lead to superior piezoelectric outputs.

### 3.3. Nanotree

**Figure 3:**
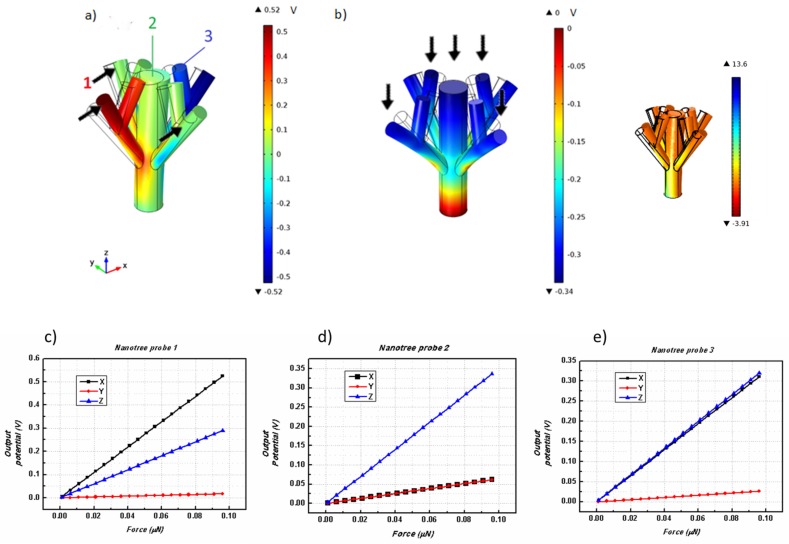
Nanotree structural deflection and corresponding piezoelectric potential when applying (**a**) *F*_x_, and (**b**) *F*_z_, on the top surface. Inset shows the calculated stress for a force applied along the *x*-direction. The *c*-axis is directed along the long nanowire direction of each tree branch. Resulting piezoelectric output potentials as a function of the total applied force along the *x*-, *y*- and *z*-directions for (**c**) probe 1, (**d**) probe 2, and (**e**) probe 3, respectively. The location of the measurement probes 1, 2, and 3 are represented in (**a**). A color scale in Volts shows the piezoelectric potential distribution on the structure.

To improve both perpendicular and parallel energy conversions through applied forces, we created and simulated the behavior of a nanoscaled tree ZnO structure. Starting from a similar nanowire (*H* = 500 nm, *R* = 50 nm) now acting as a “trunk”, we attached large “branches” (with *R* = 25 nm) that were 333.33 nm in length, and then, attached shorter “branches” that were 166.66 nm in length to the larger branches (Figure 3). To effectively measure the output potential in this complex structure, three different probes were used, one on the top surface of the nanowire “trunk”, and the other two on the top surface of the “branches” with different lengths.

The simulation results are illustrated in Figure 3. Figure 3a shows the piezoelectric potential for a force applied along the *x*-direction. In this case, one can observe that the largest potential variations occur near the intersection of the branches where the imposed curvature is stronger. The piezoelectric potential also has an anti-symmetry axis relative to the middle of the main wire. For a force applied along the *z*-direction (Figure 3b), the piezoelectric potential has its largest variations near the anchor points of the structure. Again, the stress results correlate with those obtained for the piezoelectric potential. The stress is symmetrical along the nanotree main trunk, being larger at the fixed base and at the branch intersection points. Analyzing Figure 3, one can clearly see the improvement in the lateral forces’ performance, with the value of the output voltage increasing three times from the previous setups, reaching a maximum potential of 0.52 V. However, for a perpendicular applied force (along the *z*-axis), the obtained value is almost half of the one simulated for a single cylindrical nanowire.

The results plotted in Figure 3c–e show that there is a large potential that arises from using a ZnO nanotree-like shape for laterally configured NGs that utilize a difference in potential between the opposite sides of the tree. However, smaller output potential values were obtained under perpendicular compressive forces. Due to its spread surface area, this nanotree structure is more susceptible to deformations than a simple nanowire, further increasing the sensitivity of the system, which is an important factor for more accurate and sensitive piezoelectric sensors.

### 3.4. Discussion

**Figure 4:**
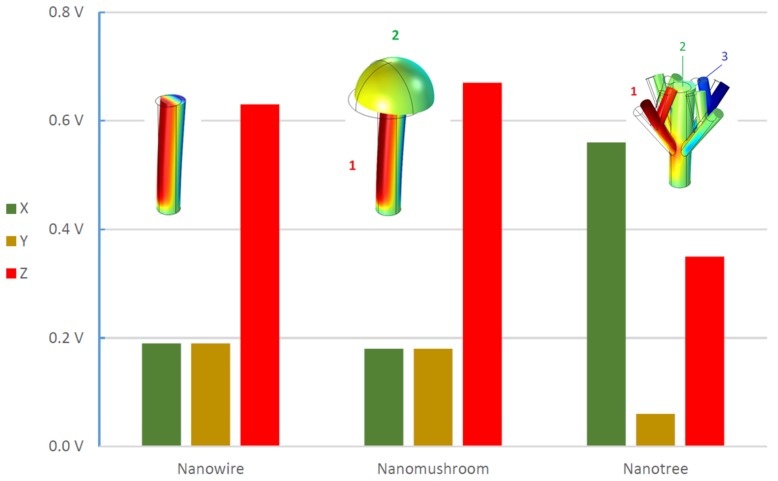
Piezoelectric potential maximum values for nanowire, nanomushroom and nanotree structures applied along the *x*-, *y*-, and *z*-axis.

Figure 4 and Table 2 illustrate the comparison between the piezoelectric potentials obtained for forces applied in the *x*-, *y*-, and *z*-directions for the different nanostructures. From these, one sees that, for the same force magnitude, forces applied along the *z*-direction always lead to higher piezoelectric potentials in non-branched structures, with the nanowire and nanomushroom designs giving similar output values. Furthermore, the displacement is always smaller for *z*-direction forces when compared with those applied along the other two directions, which resulted in a much larger displacement as seen by the bending of the structures. For the branched nanotree, one can clearly see much larger output values being generated for forces applied along the *x*-direction than in the other structures. The force in the *x*-direction displaces the branches towards the central axis of the nanowire and produces maximum piezoelectric potential.

In addition, note that our simulations used the bulk piezoelectric parameters of ZnO to simulate the energy harvesting properties of ZnO nanowires. However, first principles investigations showed that the piezoelectric coefficients change at the nanoscale and are size dependent, which could lead to higher output potentials [42]. Furthermore, the effect of doping and free charge carriers in ZnO nanowires can also be implemented [16,43,44].

Several practical implementation aspects should also be considered for the actual fabrication of these structures. The dimension and position of the electrical contacts is known to be an important parameter when considering piezoelectric NGs [33,41], particularly in the case of lateral bending. The most common and practical configuration involves an electrode at the bottom and another at the top of the nanowire (or of an array of nanowires). This is also the most efficient when a compressive force is applied (along the *z*-axis) as the piezoelectric potential is generated along the long axis of the nanowire. However, for lateral bending displacements (forces applied along each of the *x*- and *y*-axes), the potential difference is created along the short nanowire axis and the electrodes should then be fabricated in opposite sides of the bottom of the nanowire above the depleted, low potential difference zone [41]. This adds challenges in the nanotree case where the maximum potential difference is found in the small branches forking from the main nanowire. Another challenge is that of creating the bending itself. For this, the use of an already implemented zig-zag, Pt-coated, silicon top electrode could be the solution [45].

## 4. Conclusions

A finite elements method allowed us to numerically simulate the effect of different deformations on piezoelectric zinc oxide nanostructures. Important relations between form, geometric parameters, and output voltage were extracted. For parallel forces (applied along each of the *x*- and *y*-axes), we observed that the output was at a minimum for the nanowire and nanomushroom structures, and that much higher output potentials were obtained in the nanotree structure. This was because, as *F*_x_ and *F*_y_ were applied in branches, they would take advantage of the *c*-axis of the branch and produce energy from the $e_{33}$ coefficient. Therefore, the most important factor to consider is to orientate the structures so they can be deformed parallel with their *c*-axis.

Thus, for perpendicular nanogenerators, simple nanowires are suitable, and the improvement by increasing complexity of the structures will be modest. However, for parallel devices, the lateral forces were found to have a weak performance on the simple nanowires, such that other designs must be applied. In this work, we showed that the nanotree-based structure ensures this objective, with highly enhanced lateral performances along both the *x-* and *y-*axis, and that they are the most efficient structures to be implemented on nanogenerators.

## Figures and Tables

**Table 1:** List of ZnO material parameters used in the simulations.

Constant	Value	Unit
***ε*_11_** **/** ***ε*_0_**	8.5	
***ε*_33_** **/** ***ε*_0_**	10.5	
***d*_33_**	12.4	10^−12^ C/N (10^−12^ m/V)
***d*_33_**	−5.0	
***d*_15_**	−8.3	
***e*_33_**	1.57	C/m^2^
***e*_33_**	−0.36	
***e*_15_**	−0.36	
***c*_33_**	210	N/m^2^
***c*_12_**	121	
***c*_13_**	105	
***c*_33_**	211	
***g*_33_**	0.13	Vm/N
***g*_31_**	−0.05	

**Table 2:** Piezoelectric potentials and nanostructures displacements.

Structure	Applied Force (μN)			Maximum Piezoelectric Potential (V)	Displacement (nm)
	*x*	*y*	*z*		
Nanowire	100			0.19	5.8
		**100**		0.19	5.8
			**100**	0.63	0.03
Nanomushroom	100			0.17 (@Probe 1)	7.0
		**100**		0.17 (@Probe 1)	7.0
			**100**	0.63 (@Probe 2)	0.04
Nanotree	100			0.52 (@Probe 1)	7.2
		**100**		0.06 (@Probe 2)	4.6
			**100**	0.34 (@Probe 2)	0.02
