# Addendum: Cardoso, J., *et al.* The Influence of Shape on the Output Potential of ZnO Nanostructures: Sensitivity to Parallel versus Perpendicular Forces. *Nanomaterials* 2018, *8*, 354

**DOI:** 10.3390/nano10061041

**Published:** 2020-05-29

**Authors:** José Cardoso, Filipe F. Oliveira, Mariana P. Proenca, João Ventura

**Affiliations:** IFIMUP-IN, and Department of Physics and Astronomy, Faculty of Sciences, University of Porto, Rua do Campo Alegre, 687, 4169-007 Porto, Portugal; filipe.f.oliveira91@gmail.com (F.F.O.); marianapproenca@gmail.com (M.P.P.)

The authors wish to add the following information to the acknowledgements section of their paper published in *Nanomaterials* [1]. 

This research was funded by national funds by Fundação para a Ciência e a Tecnologia (FCT), Foundation for Science and Technology (I.P.) through the Associated Laboratory (IN) and by European funds through Fundo Europeu de Desenvolvimento Regional (FEDER) (European Regional Development Fund), by the Operational Programme for Competitiveness and Internationalisation (COMPETE2020), under project PTDC/CTM-NAN/3146/2014 (POCI-01-0145-FEDER-016623).
